# Clinical characteristics and prognostic factors for primary pediatric and adolescent Non-Hodgkin Lymphomas of the gastrointestinal tract: a population-based study

**DOI:** 10.1186/s12957-023-03238-9

**Published:** 2023-11-15

**Authors:** Peng Wu, Dongsheng Zhu, Yi Lou, Xinxin Wang

**Affiliations:** 1 Department of Pediatric Surgery, Northwest Women’s and Children’s Hospital, Xi’an, Shaanxi, China; 2 Department of Pediatric Surgery, The First People’s Hospital of Lianyungang, Haizhou District, Lianyungang, People’s Republic of China; 3 Department of Pediatric Surgery, Hangzhou Children’s Hospital, Hangzhou, Zhejiang China; 4 Department of Radiation Oncology, The Third Hospital of Nanchang, Nanchang, 330025 Jiangxi China

**Keywords:** Pediatric, Non-Hodgkin Lymphomas, Gastrointestinal tract, Survival

## Abstract

**Purpose:**

To investigate the clinical features and survival outcomes of primary gastrointestinal non-Hodgkin lymphomas (PGINHL) in pediatric and adolescent population, we conducted a population-based cohort study.

**Methods:**

All pediatric and adolescent patients with PGINHL diagnosed between 2000 and 2019 were identified using the Surveillance, Epidemiology, and End Results (SEER) database. Kaplane-Meier estimations were used to generate survival curves based on various criteria. To compare survival curves, the log-rank test was applied. A multivariate Cox proportional hazards model was developed to investigate the effect of each component on overall survival.

**Results:**

A total of 334 pediatric and adolescent with PGINHL patients were identified. The median age at diagnosis was 12 years (range 1.0–19 years). Tumors were most commonly found in the small bowel (47.3%), followed by the large bowel (42.8%) and the stomach (9.9%). Overall, the most common histological subtype was Burkitt lymphoma (56.9%), followed by diffuse large B-cell lymphoma (DLBCL) (27.8%). Overall survival rates for all patients were 92.2% at 5- year and 91.6% at 10- year, respectively. The Cox proportional hazard regression revealed that only chemotherapy was an important independent predictor in this model. Patients with chemotherapy have a higher survival rate than those without.

**Conclusions:**

Our study revealed that only chemotherapy was found to be the most important predictor of the OS in pediatric and adolescent PGINHL, providing critical information for therapeutic care.

## Introduction

Primary gastrointestinal system tumors are uncommon in children and adolescents. Compared with colorectal and gastric carcinomas in adults, gastrointestinal lymphomas are the most prevalent primary malignancies of the gastrointestinal tract in children and are mostly non-Hodgkin lymphomas (NHL) [1]. Non-Hodgkin lymphoma (NHL) accounts for about 7–12% of all pediatric cancers, and a third of NHL cases are predominantly caused by extranodal areas [2]. In addition, pediatric and adolescent primary gastrointestinal NHL (PGINHL) occurs more frequently in the ileocecal region than in the stomach, in contrast to adult PGINHL [3].

Because of the lower overall prevalence of pediatric and adolescent PGINHL patients, most retrospective and observational studies have a fairly small number of participants, making it difficult to draw firm conclusions [4, 5]. Therefore, it is critical to investigate the factors impacting survival rates in pediatric and adolescent PGINHL separately. The Surveillance, Epidemiology, and End Results (SEER) database was used to investigate all pediatric and adolescent PGINHL diagnosed between 2000 and 2019. Furthermore, we compared survival rates to explore the impact of surgery and chemotherapy.

## Methods

### Study population

The current population-based analysis was derived from the National Cancer Institute's (NCI) Surveillance, Epidemiology, and End Results (SEER) program (www.seer.cancer.gov). The data for the study were extracted from the SEER 18 registries database using the Surveillance Research Program, National Cancer Institute SEER*STAT software version 8.4.1, which was used to search for cases of pediatric and adolescent PGINHL using the International Classification of Diseases for Oncology (ICD-O-3) (9687/3: Burkitt lymphoma, 9680/3: Diffuse large B-cell lymphoma (DLBCL), and 9591/3: Non-Hodgkin lymphoma, NOS). The study only included pediatric and adolescent PGINHL cases diagnosed between 2000 and 2019. Informed consent or an ethical review were not necessary because the patients' private information could not be recognized in our study.

The database was used to collect information about the patient's age at diagnosis, gender, race, tumor histology, primary site, SEER stage, chemotherapy, surgery, survival status, and survival months. The SEER Summary stage categorization was completed in accordance with the 2018 SEER Summary Stage Coding Instructions. The age at diagnosis was divided into two categories: 10-year-old or younger and older than 10-year-old. The races were classified into three groups: white, black, and others. Primary sites include the small bowel, large bowel, and stomach. There are four types of varied treatment: no treatment, surgery alone, chemotherapy alone, and surgery plus chemotherapy. The primary outcome measure was overall survival (OS). The survival period was computed from the date of diagnosis to the most recent follow-up date, or until death.

### Statistical analysis

SPSS software (version 22.0, SPSS Inc., Chicago, IL, USA) was used for statistical analysis. The Kolmogorov–Smirnov test was employed to assess the distribution's normality. For data that was normally distributed, the Student's t test was applied. For the purpose of comparing non-normal data distributions, the Kruskal–Wallis test was used. Categorical variables were compared using the Chi-square test. The 5- and 10-year overall survival rates, computed by the Kaplan–Meier method, were examined using the log-rank procedure. The statistical significance was determined using a two-tailed *P*-value of 0.05.

## Results

### Patient characteristics

A total of 334 qualifying instances of PGINHL in pediatric or adolescent patients were identified. The median age at diagnosis was 12 years (range 1.0–19 years). Of the 334 patients, 146 (43.7%) and 188 (56.3%) were 10 years or younger and older than 10 years, respectively. The tumors primarily affected whites and males (79.6% and 78.7%, respectively). Overall, Burkitt lymphoma (56.9%) was the most prevalent histological subtype, followed by diffuse large B-cell lymphoma (DLBCL) (27.8%). Tumors were most commonly found in the small bowel (47.3%), followed by the large bowel (42.8%) and the stomach (9.9%). Patients in our study most frequently had localized (41.9%) and regional disease (36.2%), and most of them received surgery (71.0%) and chemotherapy (88.3%). Furthermore, surgery plus chemotherapy was the most widely employed treatment strategy (66.5%).

Table 1 contains a list of the 334 patients with PGINHL from diverse origins. White (82.2%), young children (0–10 years old) (48.7%), DLBCL (30.4%), and surgery (81.6%) had the highest percentages in small bowel origins. Additionally, PGINHL of large bowel origins had the largest proportions in male (83.9%), Burkitt (65.7%), and chemotherapy (95.8%), whereas the proportion of no treatment (2.1%) and surgery alone (2.1%) was the lowest. In comparison to small and large bowel origins, stomach origins had the lowest proportion of chemotherapy (54.5%) but the largest percentage of distant stage (42.5%), and no treatment (42.5%).

**Table 1:** Basic characteristics of patients stratified by tumor site

Features	Small bowel		Large bowel		Stomach		All	
	N	%	N	%	N	%	N	%
All	158	47.3	143	42.8	33	9.9	334	100
Gender								
Male	125	79.1	120	83.9	18	54.5	263	78.7
Female	33	20.9	23	16.1	15	45.5	71	21.3
Age at diagnosis (years)								
≤ 10	77	48.7	61	42.7	8	24.3	146	43.7
> 10	81	51.3	82	57.3	25	75.7	188	56.3
Ethnicity								
White	130	82.2	113	79.0	23	69.7	266	79.6
Black	15	9.5	16	11.2	3	9.1	34	10.2
Others	13	8.3	14	9.8	7	21.2	34	10.2
Tumor histology								
Burkitt	87	55.1	94	65.7	9	27.3	190	56.9
DLBCL	48	30.4	36	25.2	9	27.3	93	27.8
Others	23	14.5	13	9.1	15	45.4	51	15.3
SEER Stage								
Localized	66	41.8	60	42.0	14	42.4	140	41.9
Regional	61	38.6	55	38.5	5	15.1	121	36.2
Distant	31	19.6	28	19.5	14	42.5	73	21.9
Surgery								
Yes	129	81.6	106	74.1	2	6.0	237	71.0
No	29	18.4	37	25.9	31	94.0	97	29.0
Chemotherapy								
Yes	140	88.6	137	95.8	18	54.5	295	88.3
No	18	11.4	6	4.2	15	45.5	39	11.7
Treatment regimens								
No treatment	7	4.3	3	2.1	14	42.5	24	7.2
Chemotherapy alone	22	14.0	34	23.8	17	51.5	73	21.8
Surgery alone	11	7.0	3	2.1	1	3.0	15	4.5
Chemotherapy + surgery	118	74.7	103	72.0	1	3.0	222	66.5

### Survival and prognosis analysis

**Fig. 1:**
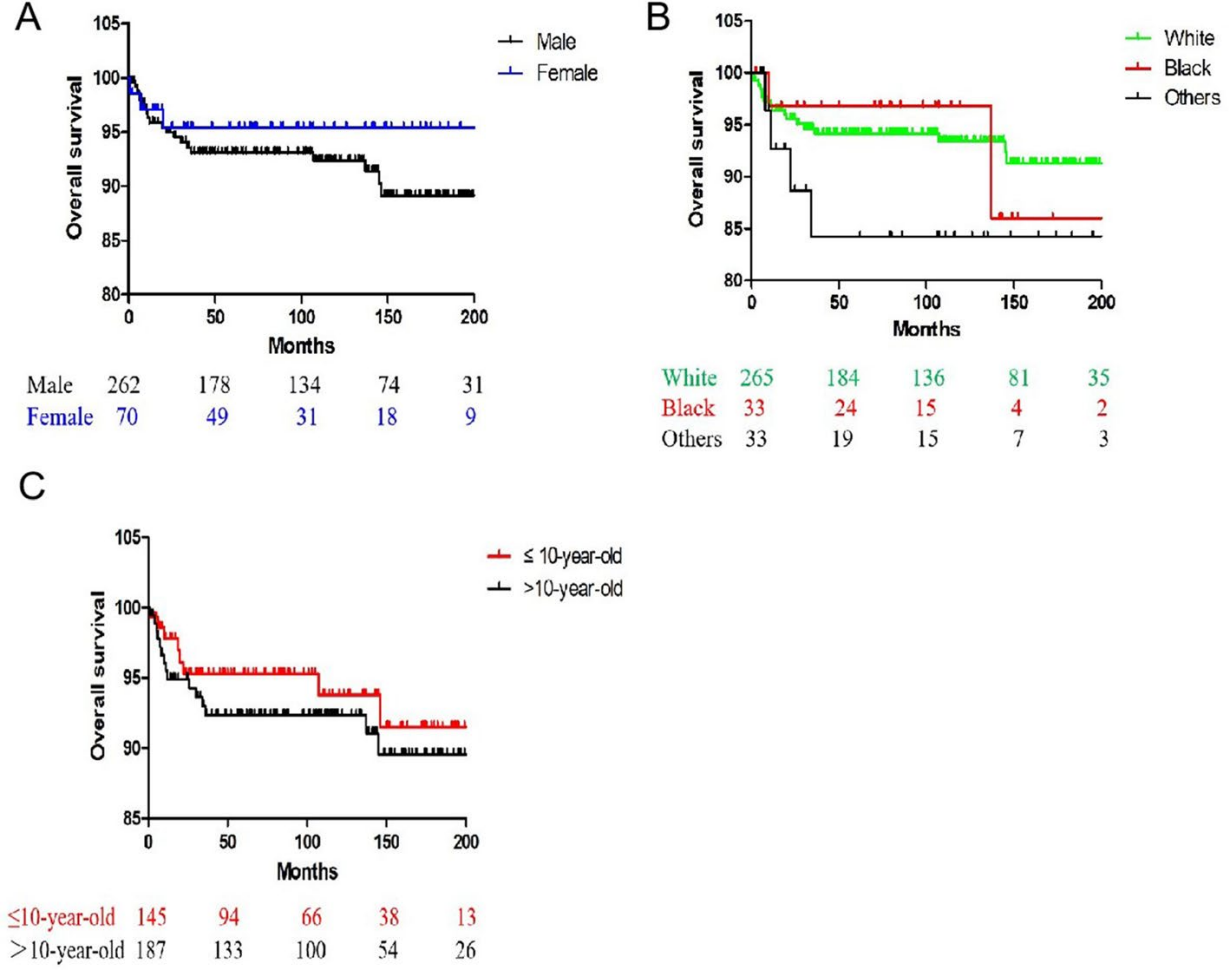
Kaplan–Meier analysis of OS among children with PGINHL, when stratified by gender, race, and age at diagnosis. **A** Female vs. male, *P* = 0.376. **B** White vs. Black vs. others, *P* = *0.607*. **C** ≤ 10-year-old vs. > 10-year-old, *P* = *0.261*

**Fig. 2:**
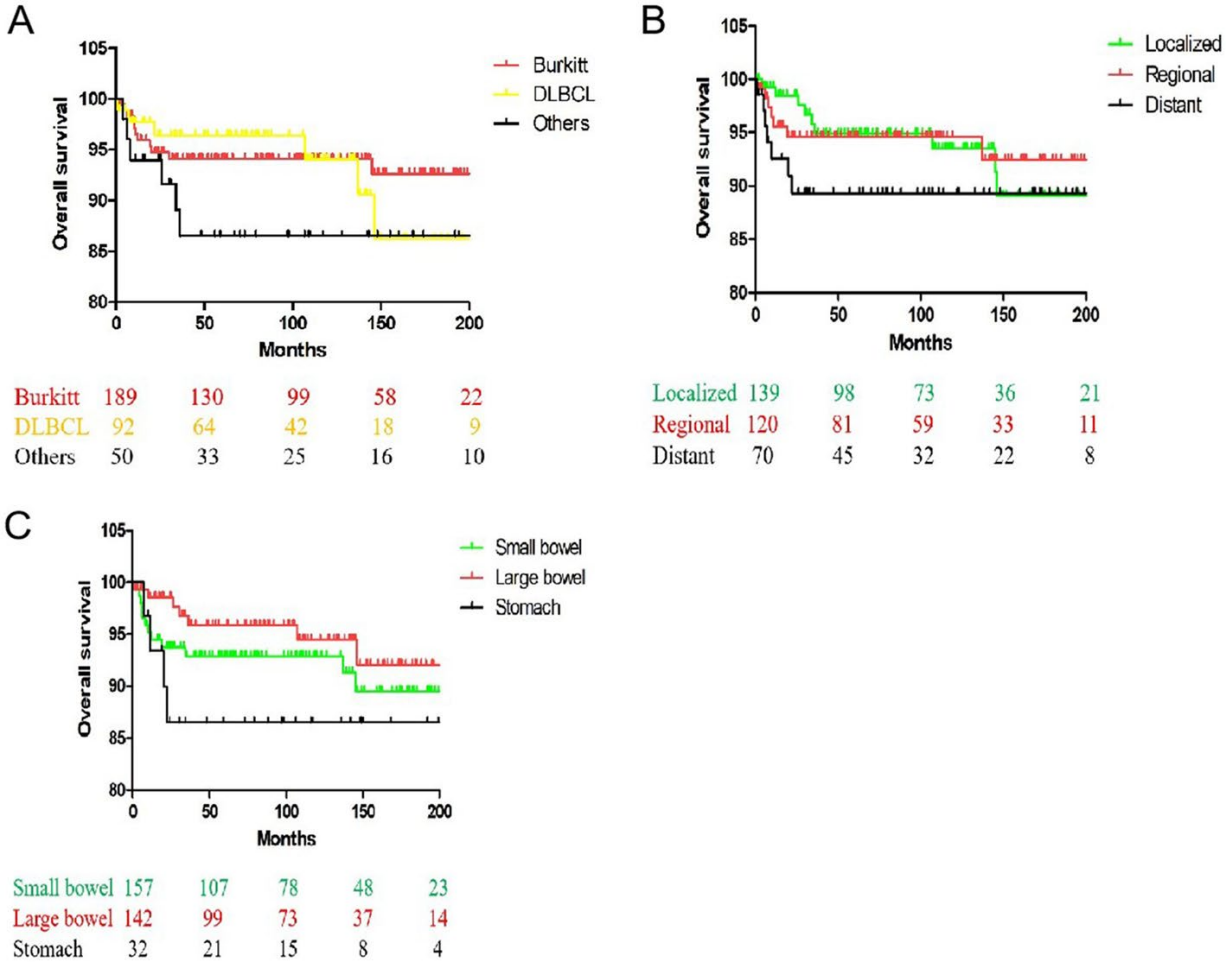
Kaplan–Meier analysis of OS among children with PGINHL, when stratified by tumor histology, SEER stage and primary sites. **A** Burkitt vs. DLBCL and Others, *P* = 0.477. **B** Distant vs. Localized and Regional, *P* = *0.277*. **C** Stomach vs. Small bowel and Large bowel, *P* = *0.041*

**Fig. 3:**
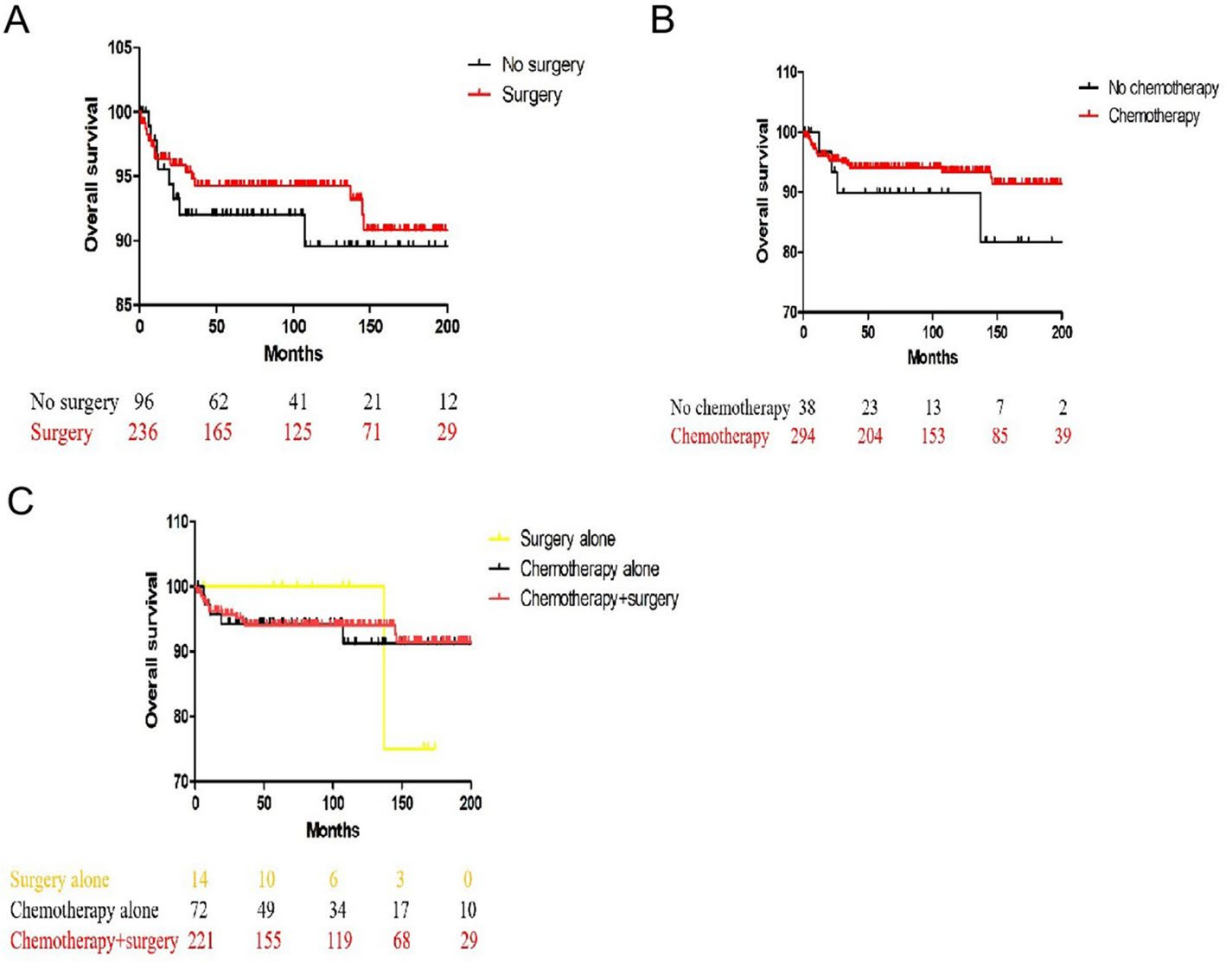
Kaplan–Meier analysis of OS among children with PGINHL, when stratified by surgery, chemotherapy, and different treatment regimens. **A** Surgery vs. No surgery, *P* = 0.399. **B** Chemotherapy vs. No chemotherapy, *P* < *0.001*. **C** Chemotherapy plus surgery and chemotherapy alone significantly improved the OS rate, *P* = 0.001

Overall 5-year and 10-year survival rates for all patients were 92.2% and 91.6%, respectively. By gender, race, and age at diagnosis, there were no significant differences in the 5-year overall survival rates (*P* = 0.376, *P* = 0.607, and *P* = 0.261, respectively) (Table 2) (Fig. 1A, 1B, and 1C). Also, there was no statistically significant difference in the overall survival by tumor histology or SEER stage for all patients (*P* = 0.477, *P* = 0.277, respectively) (Table 2) (Fig. 2A, 2B). By location, the overall survival rates of children with small and large bowel origin NHL were similar and significantly better than those of patients with stomach origin NHL (*P* = 0.041) (Table 2) (Fig. 2C). There was no significant difference in survival between those who had surgery and those who did not (*P* = 0.399) (Fig. 3A). In order to study the role of surgery in localized disease, we performed a subgroup analyses and found no significant difference in the survival for all localized patients (*P* = 0.458). By examining the survival curve, we found that patients who received chemotherapy had a significantly higher chance of survival than those who did not (*P* < *0.001*) (Fig. 3B). Chemotherapy-based regimens outperformed other regimens in terms of survival rates (*P* = *0.001*) (Fig. 3C).

**Table 2:** 5- and 10-year survival for entire cohort and by subgroup

Feature	5-Year OS (standard error) (%)	10-Year OS (standard error) (%)	*p*
Overall	92.2 (1.5)	91.6 (1.6)	
Gender			0.376
Male	91.7 (1.8)	91.0 (1.9)	
Female	94.0 (2.9)	94.0 (2.9)	
Race	0		.607
White	92.4 (1.7)	91.7 (1.8)	
Black	96.8 (3.2)	96.8 (3.2)	
Others	84.3 (7.3)	84.3 (7.3)	
Age at diagnosis			0.261
≤ 10-year-old	94.6 (2.0)	90.9 (3.3)	
> 10-year-old	90.4 (2.2)	89.1 (2.5)	
Primary sites			0.041
Small bowel	91.1 (2.4)	91.1 (2.4)	
Large bowel	95.9 (1.8)	94.5 (2.3)	
Stomach	81.3 (6.9)	81.3 (6.9)	
Tumor histology			0.477
Burkitt	93.1 (1.9)	93.1 (1.9)	
DLBCL	93.3 (2.6)	91.0 (3.4)	
Others	86.6 (5.2)	86.6 (5.2)	
SEER stage			0.277
Localized	94.9 (2.0)	93.5 (2.4)	
Regional	93.1 (2.4)	93.1 (2.4)	
Distant	86.3 (4.3)	86.3 (4.3)	
Surgery			0.399
Yes	93.1 (1.7)	93.1 (1.7)	
No	90.1 (3.1)	87.7 (3.8)	
Chemotherapy			< 0.001
Yes	94.0 (1.5)	93.4 (1.6)	
No	78.4 (6.8)	78.4 (6.8)	
Treatment regimens			0.001
Chemotherapy alone	94.3 (2.8)	91.2 (4.0)	
Surgery alone	80.0 (10)	80.0 (10)	
Chemotherapy + surgery	94.0 (1.7)	94.0 (1.7)

Table 3 displays the findings from a Cox proportional hazards model analysis involving 334 patients with PGINHL. In this model, only chemotherapy was an important independent predictor of OS, according to the results of Cox proportional hazard regression. Patients who did not receive chemotherapy had a higher chance of death (hazard ratio (HR) 4.74, 95% confidence interval (CI), 1.55–14.5; *P* = 0.006). The risk of death from stomach-originating NHL was not significantly higher than that from intestinal-originating NHL (HR, 0.68; 95% CI, 0.17–2.68; *P* = 0.580).

**Table 3:** Cox proportional hazards multivariable regression for children and adolescents with primary gastrointestinal Non-Hodgkin lymphomas

Variables	Hazard ratio (95% CI)	*P*
Primary sites		
Small bowel	0.68 (0.17–2.68)	0.580
Large bowel	0.38 (0.09–1.63)	0.195
Stomach	Reference	
Chemotherapy		
No	4.74 (1.55–14.5)	0.006
Yes	Reference	
Treatment regimens		
Chemotherapy + surgery	0.67 (0.36–0.95)	0.031
Chemotherapy alone	0.54 (0.26–0.82)	0.039
Surgery alone	Reference	

## Discussion

Primary gastrointestinal lymphomas (PGL) account for the majority of gastrointestinal extranodal lymphomas in adults, with intestinal lymphomas ranking second [6, 7]. In contrast, the intestine is the most commonly afflicted site in children and adolescents, whereas the stomach accounts for only 10% of gastrointestinal lymphomas [8]. It is well known that stomach lymphoma is largely an adult illness, peaking in incidence between the sixth and seventh decades of life [7]. DLBCL is more common in adults, whereas Burkitt lymphoma is the most common histological type in children and adolescents. Additionally, a higher percentage of the pediatric group underwent surgical resection than that of the adult group.

In this study, we presented one of the largest datasets to explore the role of surgical resection and chemotherapy treatment in pediatric and adolescent PGINHL. Our research verified what had already been documented, namely that Burkitt lymphoma was the primary histological subtype of these tumors and that most of them were found in the small bowel. Although boys are more likely than girls to develop malignancies, our research found no difference in survival rates between the genders. Except for stomach NHL, which appears with distant disease in 42.5% of cases, most of these tumors develop at the loco-regional stage. However, no significant survival difference was found between localized and distant stages. Tumor location was found to be a reliable predictor of survival in the univariate analysis. However, primary sites had no significant relationship with survival in the multivariate model. We also found no significant difference in survival between those who had surgery and those who did not, which is in line with earlier published reports on pediatric and adolescent PGINHL [9].

Over the years, the role of surgery in the treatment of pediatric abdominal lymphoma has changed substantially. Historically, the majority of PGL treatment was surgical excision, followed by postoperative chemotherapy. Series from the early 1990s indicated the survival advantage of near-complete excision of the original tumor in patients with abdominal lymphoma [10–12]. Due to improved treatment protocols, debulking surgery is no longer advocated, and surgical resection is now only recommended for localized disease, or individuals presenting with intestinal obstruction [13, 14]. However, it was discovered after the 1990s that rigorous chemotherapy alone was successful for high-grade stomach lymphoma [15]. The outcome for advanced stage patients without major surgical resection has improved with current treatment methods [16]. Chemotherapy was found to be the most effective intervention in our study for increasing survival. 91.5% of individuals with PGINHL underwent chemotherapy. The absence of chemotherapy has become the most significant factor influencing survival. The chemotherapy group had a 5-year and 10-year OS of 94% and 93.4%, respectively, which was considerably greater than the no-chemotherapy group (78.4% for both the 5-year and 10-year OS). Furthermore, the Cox proportional hazard regression demonstrated that only chemotherapy considerably reduced the chance of death.

The current study has a number of potential limitations that should be considered. Firstly, a lot of specific clinical data was not documented, including diagnostic techniques, chemotherapy regimens, and immunotherapy. Secondly, although data on the receipt of surgery was available, information about about the types of surgery (biopsy or tumor resection) was not available. Finally, the patients in the SEER database are mostly white or black, which may limit the conclusion's application to children of other races. A future study is therefore necessary to confirm the findings.

In conclusion, we used a population-based dataset to evaluate the clinical features and prognosis of pediatric and adolescent PGINHL. Chemotherapy was discovered to be the most important predictor of survival. Our findings showed no advantage of surgical resection, reiterating the importance of chemotherapy as the main treatment methods.

## Data Availability

The dataset used and analyzed during the current study are available from the corresponding author on reasonable request.
